# Noise exposure in occupational setting associated with elevated blood pressure in China

**DOI:** 10.1186/s12889-017-4050-0

**Published:** 2017-01-23

**Authors:** Shuchang Chen, Yaqin Ni, Lei Zhang, Liya Kong, Luying Lu, Zhangping Yang, Luoxian Yang, Xuhui Zhang, Yimin Zhu

**Affiliations:** 1 Hangzhou Center for Disease Control and Prevention, Hangzhou, 310021 People’s Republic of China; 2 Department of Epidemiology and Biostatistics, Zhejiang University School of Public Health, 388 Yu-Hang-Tang Road, Hangzhou, Zhejiang Province 310058 People’s Republic of China; 3 Hangzhou Hospital for Prevention and Treatment of Occupational Diseases, Hangzhou, Zhejiang Province 310014 People’s Republic of China; 4 Department of Epidemiology and Biostatistics, Zhejiang Chinese Medical University, Binwen Rd 548, Hangzhou, Zhejiang Province 310053 People’s Republic of China

**Keywords:** Occupational noise exposure, Hypertension, Systolic blood pressure (SBP), Diastolic blood pressure (DBP), Odds ratio (OR)

## Abstract

**Background:**

Hypertension is the primary out-auditory adverse outcome caused due to occupational noise exposure. This study investigated the associations of noise exposure in an occupational setting with blood pressure and risk of hypertension.

**Methods:**

A total of 1,390 occupational noise-exposed workers and 1399 frequency matched non-noise-exposed subjects were recruited from a cross-sectional survey of occupational noise-exposed and the general population, respectively. Blood pressure was measured using a mercury sphygmomanometer following a standard protocol. Multiple logistic regression was used to calculate the odds ratio (OR) and 95% confidence interval (CI) of noise exposure adjusted by potential confounders.

**Results:**

Noise-exposed subjects had significantly higher levels of systolic blood pressure(SBP) (125.1 ± 13.9 mm Hg) and diastolic blood pressure (DBP) (77.6 ± 10.7 mm Hg) than control subjects (SBP: 117.2 ± 15.7 mm Hg, DBP: 70.0 ± 10.5 mm Hg) (*P* < 0.001). Significant correlations were found between noise exposure and blood pressure (SBP and DBP) (*P* < 0.001). However, the linear regression coefficients with DBP appeared larger than those with SBP.

The prevalence of hypertension was 17.8% in subjects with noise exposure and 9.0% in control group (*P* < 0.001). Compared with the control group, the subjects with noise exposure had the risk of hypertension with an OR of 1.941 (95% CI = 1.471– 2.561) after adjusting for age, sex, smoking, and drinking status. Dose–response relationships were found between noise intensity, years of noise exposure, cumulative noise exposure and the risk of hypertension (all *P* values < 0.05). No significant difference was found between subjects wearing an earplug and those not wearing an earplug, and between steady and unsteady noise categories (*P* > 0.05).

**Conclusions:**

Occupational noise exposure was associated with higher levels of SBP, DBP, and the risk of hypertension. These findings indicate that effective and feasible measures should be implemented to reduce the risk of hypertension caused by occupational noise exposure.

## Background

Noise is the most common occupational risk factor and millions of workers are exposed to harmful levels of noise in the workplace [1, 2]. Hearing loss is a well-documented primary biological adverse effect caused by occupational noise exposure [3, 4]. In addition, noise exposure activates the sympathetic and endocrine systems, thereby affecting the humoral and metabolic states of human beings [5–8]. Therefore, noise exposure increases the risk of out-auditory adverse outcomes such as hypertension and cardiovascular diseases, digestive and behavioral disorders, and sleep disturbances [7, 9, 10].

Although the associations between noise exposure and blood pressure and/or hypertension have been extensively studied [11–16], however, the findings are not always consistent. Previous studies have primarily focused on community noise exposure [14, 17–19]. There were large variations in the characteristics of noise exposure between community and occupational exposure in terms of intensity, duration, and category of noise exposure. Previous findings in occupational settings were not always consistent. Significant positive associations were found in cohort study [12, 20, 21] and cross-sectional studies [22, 23]. However, negative associations were also found in other studies [24, 25]. These inconsistent findings might be attributed to the study design, population, exposure evaluation, and modification of potential confounding factors. Different types of noise exposure and measurements of protection would affect the biological functions and cause blood pressure and cardiovascular diseases.

The objective of this study was to evaluate the associations of occupational noise exposure with blood pressure and hypertension in the Chinese population.

## Methods

### Subjects

The subjects in this study included 1390 occupational noise exposed workers (exposed group) and 1399 non- noise-exposed subjects (control group). The exposed subjects were randomly recruited from a cross-sectional survey of occupational noise exposure in Hangzhou, Zhejiang Province, China, which has been previously described in detail [26]. In that cross-sectional survey, the subjects were the workers who were employed in the noise-exposed factories of mechanical equipment and household appliance manufacturing, steel construction, and cigarette production/packaging in Hangzhou city, Zhejiang province, China. Subjects had occupational noise exposure for more than one year and the intensity of noise exposure was >80 dB (A) (L_EX,8h_). The workers were excluded if they had hypertension and other chronic diseases such as coronary heart disease, cancer, and kidney diseases. Before noise exposure. Epidemiological data were collected by face to face interview using a structured questionnaire and administered by trained professional physicians. The information in the questionnaire included demographic characteristics, smoking/drinking status, history of medical conditions and drug use, history of exposure to noise, vibration, and toxic chemicals in the workplace, health habits, and use of ear protection for noise. Intensity of noise in the workplace was determined by a noise statistical analyzer (AWA6218; Westernization Instrument Technology Co., Ltd., Beijing, China). Noise exposure was evaluated with equivalent continuous dB(A)- weighted sound pressure levels (LEX,8 h) according to the Occupational Health Standard of the People’s Republic of China: Measurement of Noise in the Workplace (GBZ/T 189.8–2007) (China, 2007). Because the majority of subjects in the cross-sectional study were males (about 91.7%), the subjects in the present study were restricted to males.

Control subjects were recruited from a cross-sectional survey of general population on metabolic syndrome in the same area with occupational noise survey. In this survey, blood pressure were measured and epidemiological data were collected using the same methods as for the exposed group. Control subjects had no specific noise exposure in workplace and resident area, which met the hygienic standard for noise in industrial enterprises and social living environmental noise (GB 3096–2008 and GB 22337–2008). The control subjects were frequency-matched with the exposed groups in the distribution of age, sex, and resident area. The study protocol was approved by the Research Ethics Committees of Hangzhou Center for Disease Prevention and Control, Zhejiang, China.

### Blood pressure measurement and hypertension definition

Following gap of more than 12 h after noise exposure, systolic blood pressure (SBP) and diastolic blood pressure (DBP) were measured by trained physicians following a standard protocol. Blood pressure was measured using a mercury sphygmomanometer with subjects in the sitting position after more than 15-min rest. SBP and DBP were reported as the average of three repeat measurements with 30-s intervals.

Hypertension was defined as SBP ≥ 140 mmHg and/or a DBP ≥ 90 mmHg. Any subject reporting the use of antihypertensive medications were also classified as hypertensive, regardless of the measured levels of blood pressure.

### Statistical analysis

Cumulative noise exposure (CNE) was calculated as CNE = 10 × log(10^SPL/10^ × years of noise exposure), where SPL is the sound pressure level [dB (A)] of noise exposure. Continuous variables for normal distribution were expressed as mean ± standard deviation (SD) and as median (P25, P75) for skewed distribution. Categorical variables were expressed as frequencies (%).

*χ* ^2^ test was used to examine the statistical significance of categorical variables., One-way ANOVA was used for continuous variables followed by multiple comparisons with SNK. Multiple logistic regression was used to compare the differences among subjects with different noise exposure levels after adjusting for confounders such as age, sex, smoking, and drinking status. Odds ratio (OR) and its 95% confidence interval (CI) were calculated for the risk of hypertension by noise exposure, adjusting for potential confounders with reference to the control group, and subjects with lowest levels of noise exposure. All statistical analyses were performed using SPSS 19.0 for Windows (IBM Corporation, Armonk, NY, US).

## Results

### Basic characteristics of the subjects

This study recruited 1,390 noise-exposed workers and 1399 non-noise exposed control subjects. The basic characteristics of the subjects are shown in Table 1. All the subjects were males with a mean of age was 33.1 years old ± 8.7 (standard deviation). No significant difference was found between the exposed and control groups in term of age (*P* > 0.05). However, 56.2% of exposed group were smokers and 46.6% in control group (*P* <0.001), and 68.6% of the exposed group were alcohol drinkers and 41.0% in control group (*P* <0.001). Therefore, smoking and drinking status were modified in the following analysis. The median of intensity of noise exposure was 87.5 dB (A) (82.5, 87.5) and the duration of noise exposure was 4.21 (1.67, 8.00) years, with a CNE value of 92.28 (88.31, 96.79). 83.8% of the exposed workers used earplug as simple noise protection.

**Table 1:** Basic characteristics of noise- exposed and control group

variables	Noise-exposed group (*n =* 1390)	control group (*n =* 1399)	*P* value
Age (years) (mean ± SD)	33.1 ± 8.7	33.1 ± 8.6	0.941^#^
Smoking status (%)			
Yes	56.2	46.6	
No	43.8	53.4	<0.001^§^
Drinking (%)			
Yes	68.6	41.0	
No	31.4	59.0	<0.001^§^
Years to noise exposure, years,*	4.2(1.7,8.0)		
Noise intensity exposure, dB (A),*	87.5 (82.5,87.5)		
cumulative noise exposure (CNE)*	92.3(88.3,96.8)		
Noise protection (%)	83.8		

CNE, cumulative noise exposure, was calculated as CNE = 10 × log 10^SPL^ × years of noise exposure), where, SPLis the sound pressure level [dB (A)] of noise exposure

*Data are presented as median (P25, P75)

^#^independent *t*-test; §, *χ* ^2^ test

### Effect of noise exposure on the levels of SBP and DBP

The levels of SBP and DBP of the subjects of the noise exposed and non-exposed groups are shown in Table 2. The mean levels of SBP in exposed group were 125.1 ± 13.9 mm Hg and 117.2 ± 15.7 mm Hg in the control group (*P* < 0.001). Exposed group had significantly higher levels of DBP than control group (*P <* 0.001). Significant differences were also found among the subgroups of exposed subjects classified by different noise intensity, duration, and CNE of noise exposure. The regression coefficients of noise exposure (different intensities, duration, and CNE of noise exposure) with SBP and DBP are shown in Table 3. Significant correlations were found between noise exposure (different intensity, duration and CNE of noise exposure) and blood pressure (SBP and DBP) (*P* < 0.001). However, the regression coefficients with DBP appeared larger than those with SBP (Table 3).

**Table 2:** Levels of SBP and DBP in the subjects of noise exposed and non-exposed groups

Groups	n	SBP		DBP	
		Mean ± SD	*P* value*	Mean ± SD	*P* value*
Non-exposed group	1399	117.2 ± 15.7		70.0 ± 10.5	
Exposed group	1390	125.1 ± 13.9	<0.001	77.6 ± 10.7	<0.001
Noise intensity (dB)					
80-	490	124.2 ± 14.7^#^		75.8 ± 11.1^#^	
85-	571	124.9 ± 12.8^#^		78.1 ± 10.1^#,$^	
90-	237	126.2 ± 14.6^#^	<0.001	78.8 ± 11.1^#,$^	<0.001
≥ 95	92	129.1 ± 13.8^#,$,§^		80.7 ± 9.5^#,$^	
Work-years of noise exposure					
1-	795	123.9 ± 13.4^#^		76.0 ± 10.3^#^	
5-	394	125.7 ± 13.8^#,$^		78.8 ± 10.3^#,$^	
10-	100	126.8 ± 13.8^#,$^	<0.001	81.5 ± 11.5^#,$,§^	<0.001
≥ 15	101	130.7 ± 16.5^#,$,§^		81.3 ± 11.1^#,$,§,§^	
cumulative noise exposure					
80-	479	123.3 ± 12.9^#^		75.2 ± 10.1^#^	
90-	434	124.8 ± 13.9^#^		77.5 ± 10.5^#,$^	
95-	274	126.0 ± 15.1^#,$^	<0.001	79.3 ± 11.5^#,$,§^	<0.001
≥ 100	203	128.8 ± 13.9^#,$,§,^&		81.0 ± 9.8^#,$,§^	
Noise category					
Stable	451	124.7 ± 12.5		77.6 ± 9.2	
Unstable	939	125.4 ± 14.5	0.369	77.6 ± 11.3	0.958
Noise protection					
Yes	956	124.4 ± 12.0		77.3 ± 9.3	
No	185	124.6 ± 13.7	0.855	77.3 ± 10.8	0.983

*P values were calculated by one-way ANOVA, and then followed by SNK for multiple comparison. #significant difference with non-exposed group. $, significant difference with the group of 80- for Noise intensity, 1- for Work-years of noise exposure, and 80- for cumulative noise exposure. §, significant difference with the group of 85- for Noise intensity, 5- for Work-years of noise exposure, and 90- for cumulative noise exposure. &, significant difference with the group of 90- for Noise intensity, 10- for Work-years of noise exposure, and 95- for cumulative noise exposure

**Table 3:** Regression coefficient of SBP and DBP with noise exposure in the noise exposed subjects

	SBP		DBP	
	beta	*P* value	beta	*P* value
Noise intensity (dB)	0.107	<0.001	0.149	<0.001
Work- years of noise exposure	0.100	0.005	0.113	0.001
Cumulative noise exposure	0.128	<0.001	0.187	<0.001

When noise exposure was categorized as steady and unsteady noise, no significant associations of SBP and DBP were found between the steady and unsteady noise categories and between subjects wearing an earplug and those not wearing an earplug.

### Effect of noise exposure on the risk of hypertension

Table 4 presents the risks of hypertension in the subjects with occupational noise exposure. The prevalence of hypertension was 17.8% in the exposed group and 9.0% in the control group (*P <* 0.001), respectively. Compared with the control group, the subjects with noise exposure had the risk of hypertension with an OR of 1.941 (95% CI = 1.471– 2.561) after adjusting for age, smoking, and drinking status. Dose–response relationships were found between noise intensity, years of noise exposure, CNE, and the risk of hypertension (all *P* values < 0.05). Similar results were found when the lowest noise exposure levels in the exposed group was considered as a reference. Consistent results were found after stratifying analyses by age, smoking and drinking status (Data not shown).

**Table 4:** the risks of hypertension in the subjects with occupational noise exposure

Groups	n	Non-hypertension	hypertension	OR1 (95%CI)*	*P* value	OR2 (95%CI)**	*P* value
Non-exposed group	1399	1273(91.0%)	126(9.0%)	1.00(Ref.)			
Noise-exposed group	1390	1143(82.2%)	247(17.8%)	1.941(1.471-2.561)	<0.001		
Noise intensity (dB)							
80-	490	411(83.9%)	79(16.1%)	1.411(0.947-2.102)	0.091	1.000(Ref)	
85-	571	471(82.5%)	100(17.5%)	2.128(1.535-2.952)	<0.001	1.510(1.005-2.268)	0.047
90-	237	193(81.4%)	44(18.6%)	2.018(1.326-3.070)	0.001	1.283(0.783-2.102)	0.323
≥95	92	68(73.9%)	24(26.1%)	3.002(1.676-5.376)	<0.001	2.018(1.068-3.811)	0.031
P_trend_					<0.001		0.064
Work- years of noise exposure							
0	1399	1273(91.0%)	126(9.0%)	1.000(Ref)			
1-	795	680(85.5%)	115(14.5%)	1.495(1.064-2.100)	0.020	1.000(Ref)	
5-	394	321(81.5%)	73(18.5%)	2.029(1.421-2.898)	<0.001	1.199(0.813-1.767)	0.360
10-	100	74(74.0%)	26(26.0%)	2.609(1.504-4.527)	0.001	1.412(0.785-2.542)	0.249
≥15	101	68(67.3%)	33(32.7%)	4.055(2.318-7.094)	<0.001	2.080(1.114-3.880)	0.021
P_trend_					<0.001		0.022
cumulative noise exposure							
≤80	1399	1273(91.0%)	126(9.0%)	1.000(Ref)			
80-	479	418(87.3%)	61(12.7%)	1.107(0.712-1.721)	0.652	1.000(Ref)	
90-	434	359(82.7%)	75(17.3%)	1.987(1.377-2.869)	<0.001	1.637(1.015-2.638)	0.043
95-	274	215(78.5%)	59(21.5%)	2.261(1.536-3.328)	<0.001	1.733(1.034-2.904)	0.037
≥100	203	151(74.4%)	51(25.6%)	2.829(1.871-4.277)	<0.001	2.006(1.154-3.488)	0.014
P_trend_					<0.001		0.018
Noise category							
Stable	451	388(86.0%)	63(14.0%)			1.000(Ref)	
Unstable	939	755(80.4%)	184(19.6%)			1.324(0.907-1.933)	0.146
Earplug wearing							
Yes	956	801(83.8%)	155(16.2%)			1.000(Ref)	
No	185	155(83.8%)	30(16.2%)			0.910(0.585-1.415)	0.674

Multiple logistic regression was used to compare the differences among the subjects with different noise exposure adjusted by confounders such as age, sex, smoking, and drinking status

*OR1 was calculated taking the non-exposed group as the reference, adjusting for age, smoking, and drinking status

**OR2 was calculated taking the lowest levels of noise exposure as the reference, adjusting for age, smoking and drinking status

No significant difference was found in the prevalence of hypertension between subjects wearing an earplug and those not wearing an earplug, and between the steady and unsteady noise categories (*P* > 0.05).

## Discussion

In this study, we investigated the associations of SBP and DBP with occupational noise exposure in 1,390 occupational noise-exposed workers and 1399 non-noise exposed control subjects. The results indicated that occupational noise exposure associated with higher levels of SBP and DBP and the risk of hypertension. Dose–response relationships were found between noise exposure and blood pressure, and the risk of hypertension.

The associations between occupational noise exposure and hypertension have been extensively investigated [6, 13, 20–25], however, the findings are still inconsistent. In this study, using frequency-matched external and internal control groups as references, we found that noise exposure elevated the blood pressure and the risk of hypertension. These findings are consistent with previous studies [13, 20, 21]. After about 10 years of follow up, Chang et al. [20] found that high noise exposure (≥85 dBA) increased the SBP of 3.2 (95% CI: 0.2 – 6.2) mm Hg and the DBP of 2.5 (95% CI: 0.1 – 4.8) (*P <* 0.05). In the present study, potential confounding factors such as age, sex, smoking, and drinking status were adjusted, and noise exposure was evaluated in term of intensity (dB(A)), years of noise exposure and CNE. The results were consistent with different levels of noise exposure. We also found a dose- response relationship between noise exposure and blood pressure (SBP and DBP)/risk of hypertension. The elevated blood pressure and the risk of hypertension even persisted at the lowest levels of noise exposure with a noise intensity of 80 – 85 dB (A), work-years of noise exposure of 1–5 years and CNE of 80–90. These findings were concordant with previous studies [20, 22]. De Souza et al. [22] found noise exposure at 75–85 dB(A) increased the risk of hypertension with an OR of 1.56 (95% CI: 1.13 – 2.17) compared with exposure at ≤ 75 dB(A). However, no increased risk of hypertension was found in the noise exposure at 80–90 dB(A) in a population study[24]. This study had relatively large sample size, however, the data were obtained from registry system. Recently, with a meta-analysis, Skogstad et al. [16] found that occupational noise exposure increased the risk of hypertension with a hazard ratio (HR) of 1.38 (95% CI:1.01–1.87).

No significant difference was found between the steady and unsteady noise exposure categories in levels of SBP, DBP and risk of hypertension. These results indicated the similar biological effects of noise exposure on blood pressure between the steady and unsteady noise. We also found no difference in the blood pressure and risk of hypertension between subjects wearing an earplug and subjects not wearing an earplug. Usually, subjects wear the earplug for noise protection, however, the result suggested low effectiveness of wearing an earplug in noise protection. This finding was similar to previous finding of noise induced-hearing loss with earplug wearing [27]. Commonly used earplugs may have a low efficiency for noise protection. On the other hand, due to discomfort, the compliance of earplug wearing among the workers is low during noise exposure in the workplace. Therefore, the low efficiency and compliance rate might lead to the decreased effectiveness of earplug wearing.

Although significant correlations of noise exposure with both SBP and DBP were observed (*P* < 0.01), however, the regression coefficients with DBP appeared larger than those with SBP. Probably, DBP is more sensitive to the reaction of noise stress than SBP. The mechanism underlying this difference needs further investigation.

The mechanism underlying the higher levels of blood pressure due to occupational noise exposure remains unclear. Acute exposure to noise is associated with short-term changes in blood pressure, heart rate, cardiac output and vasoconstriction along with increased levels of stress hormones (e.g., epinephrine, norepinephrine and corticosteroids) [16]. However, chronic and long-term noise exposure activates the sympathetic and endocrine systems, thereby affecting the humoral and metabolic states of human beings [7, 8, 10]. This could explain the higher cardiovascular risk with noise exposure. In this study, blood pressure was measured following a gap of more than 12 h after noise exposure, therefore, reflects the long-lasting biological effect.

This study had a relatively large sample size and two referent groups of external and internal control. The external control was recruited from the general population and frequency-matched with the exposed group in term of age and sex distribution. Two reference groups could increase the consistency of the results. We also adjusted for potential confounding factors such as smoking and drinking status in addition age and sex. However, there were some limitations in this study. Due to many missing data of height and weight, BMI was still not adjusted in the analysis. One limitation was environmental noise assessment. Noise exposure was evaluated with L_EX_,8 h, and not individual assessment of noise exposure. L_EX_,8 h was used according to the Occupational Health Standard of the People’s Republic of China: Measurement of Noise in the Workplace (GBZ/T189.8–2007) (China, 2007). This evaluation of noise exposure based on representative locations at the workplace at different work times and is then weighted as sound pressure levels to nominal 8 h/day. This evaluation is the routine method used for occupational noise surveillance in China. However, the evaluation might be incorrect if a worker often changes his workplace and shift time. It would be better if individual assessment of noise exposure was used. However, individual assessment of noise exposure was very difficult for field investigation with a relatively large sample. The second limitation was that the subjects were restricted to males because the majority (about 91.7%) of the noise-exposed workers in our cross-sectional study were males, which was as similar distribution of gender with the general noise-exposed workers in China. There might be a difference in the biological effects on blood pressure and risk of hypertension with respect to sex. Previous studies have indicated that females are more susceptible to occupational noise exposure than males [18, 21, 28]. Considering the gender difference, the findings in this study should be mainly focused on males. In additionally, these findings were based on a cross-sectional design and should be validated with a prospective study. The last limitation is that the subjects of external control were recruited participants from the cross-sectional survey of general population on metabolic syndrome. Although these population have no specific noise exposure in workplace and resident area, they may have reference exposure. They may also have bias with the exposed subjects group although we used frequency-match on age, sex, and resident area.

## Conclusions

In summary, the present study suggested that occupational noise exposure had higher levels of SBP and DBP and the risk of hypertension. There were dose–response relationships between noise exposure and blood pressure and the risk of hypertension. DBP appeared to be more sensitive to occupational noise exposure. These findings indicate that effective and feasible measures should be implemented to reduce the risk of hypertension and cardiovascular diseases.

## Abbreviations

CNE: Cumulative noise exposure
DBP: Diastolic blood pressure
OR: Odds ratio
SBP: Systolic blood pressure
